# A retrospective audit of active Charcot neuroarthropathy in a tertiary hospital podiatry department

**DOI:** 10.1186/1757-1146-4-S1-P30

**Published:** 2011-05-20

**Authors:** Elise J Jilbert, Deborah E Schoen, Benjamin C Trewben, Joel M Gurr

**Affiliations:** 1 Podiatry Department, Royal Perth Hospital, Perth, Western Australia, 6000, Australia; 2 Step Ahead Podiatry, Rockingham, Western Australia, 6168, Australia; 3 Clinical Senior Lecturer, Podiatric Medicine Unit, School of Surgery, University of Western Australia, Nedlands, Western Australia, 6009, Australia

## Background

Charcot Neuroarthropathy (CN) is a major foot complication of diabetes which may lead to debilitating foot deformity, recurrent ulceration and lower limb amputation. Podiatrists play a key role along the continuum of the disease from diagnosis to treatment to long-term follow-up and monitoring. The aim of this clinical audit was to gather data on patients managed with active CN in a tertiary hospital podiatry department.

## Methods

Thirty cases (26 patients) were diagnosed with active CN during the four-year period January 2005 to December 2008, and managed through the Royal Perth Hospital Podiatry Department. Data was retrospectively collected from their medical records including demographics, diabetes parameters, CN treatment and outcomes at twelve months.

## Results

Seventy three percent of cases were male. Average age 55 ± 8.6 years. Sixty percent had Type 2 insulin-requiring diabetes. Diabetes duration greater than 10 years in 83.3% cases. Diabetes control was poor (HbA1c 9 ± 1.9%). The most common locations of CN were the tarsometatarsal joints and midfoot. Seventeen percent of cases were re-activated CN. Although 40% of cases were accurately diagnosed within 4 weeks, 33% took over 2 months. Overall CN management time was 44.7 ± 25.3 weeks. Of cases (67%) which went into a total contact cast (TCC), the overall management time was 39.3 ± 29.6 weeks versus 57.5 ± 33.6 weeks for those patients who never received TCC as part of their management. Forty seven percent developed a foot ulcer within 12 months following stabilisation of their CN, and one case went on to trans-tibial amputation.

## Conclusions

These results provide an estimate for patients and practitioners as to the overall treatment time of active CN. Our findings suggest that management of CN can be prolonged, but overall treatment is reduced through the use of TCC. Despite increased awareness and education, disparity still exists for rapid diagnosis of CN. Patients are at risk of CN related foot ulceration despite close monitoring in a tertiary hospital, however, are at a relatively low risk of amputation on the short-term. The audit highlights CN is a potentially recurrent condition and patients require lifelong care and monitoring.

